# Supplementary material for: Cloud BioLinux: pre-configured and on-demand bioinformatics computing for the genomics community
Source: BMC Bioinformatics. 2012 Mar 19;13:42. doi: 10.1186/1471-2105-13-42 (PMC3372431; doi:10.1186/1471-2105-13-42)
Supplement: Additional file 1 — Supplementary 1 Cloud BioLinux software documentation in the form of a mini, self-contained website. Users need to download and uncompress the .zip file, and open through a web browser the "index.html" file available on the main directory. (ZIP 1823 kb). [file 1471-2105-13-42-S1.ZIP › Cloud-BioLinux-Package-Documentation/docs/bioconductor.html]

Bio-Linux Software Documentation Pages

Back to search form

## bioconductor

|  |  |
| --- | --- |
| Name | bioconductor |
| Description | **bioconductor** is an open source and open development software project to provide tools for the analysis and comprehension of genomic data (bioinformatics).  The project was started in the Fall of 2001. The Bioconductor core team is based primarily at the Biostatistics Unit of the Dana Farber Cancer Institute at the Harvard Medical School/Harvard School of Public Health. Other members come from various US and international institutions.  The broad goals of the projects are to:  - provide access to a wide range of powerful statistical and graphical methods for the analysis of genomic data. - facilitate the integration of biological metadata in the analysis of experimental data: e.g. literature data from PubMed, annotation data from LocusLink - allow the rapid development of extensible, scalable, and interoperable software - promote high-quality documentation and reproducible research - provide training in computational and statistical methods for the analysis of genomic data. |
| Homepage | http://www.bioconductor.org/ |
| Remote Documentation | http://www.bioconductor.org/ |

|  |
| --- |
| Please see Remote Documentation |
